# Role of Cytochrome P450 (CYP)1A in Hyperoxic Lung Injury: Analysis of the Transcriptome and Proteome

**DOI:** 10.1038/s41598-017-00516-x

**Published:** 2017-04-04

**Authors:** Krithika Lingappan, Suman Maity, Weiwu Jiang, Lihua Wang, Xanthi Couroucli, Alex Veith, Guodong Zhou, Cristian Coarfa, Bhagavatula Moorthy

**Affiliations:** 1 Department of Pediatrics, Section of Neonatology, Texas Children’s Hospital, Baylor College of Medicine, Houston, Texas USA; 2 Advanced Technology Cores, Baylor College of Medicine, Houston, Texas USA; 3 Interdepartmental Program in Translational Biology and Molecular Medicine, Baylor College of Medicine, Houston, TX USA; 4 Institute of Biotechnology, Texas A&M University Health Science Center, Houston, Texas USA

## Abstract

Hyperoxia contributes to lung injury in experimental animals and diseases such as acute respiratory distress syndrome in humans. Cytochrome P450 (CYP)1A enzymes are protective against hyperoxic lung injury (HLI). The molecular pathways and differences in gene expression that modulate these protective effects remain largely unknown. Our objective was to characterize genotype specific differences in the transcriptome and proteome of acute hyperoxic lung injury using the omics platforms: microarray and Reverse Phase Proteomic Array. Wild type (WT), *Cyp1a1*−/− and *Cyp1a2*−/− (8–10 wk, C57BL/6J background) mice were exposed to hyperoxia (FiO_2_ > 0.95) for 48 hours. Comparison of transcriptome changes in hyperoxia-exposed animals (WT versus knock-out) identified 171 genes unique to *Cyp1a1*−/− and 119 unique to *Cyp1a2*−/− mice. Gene Set Enrichment Analysis revealed pathways including apoptosis, DNA repair and early estrogen response that were differentially regulated between WT, *Cyp1a1*−/− and *Cyp1a2*−/− mice. Candidate genes from these pathways were validated at the mRNA and protein level. Quantification of oxidative DNA adducts with ^32^P-postlabeling also revealed genotype specific differences. These findings provide novel insights into mechanisms behind the differences in susceptibility of *Cyp1a1*−/− and *Cyp1a2*−/− mice to HLI and suggest novel pathways that need to be investigated as possible therapeutic targets for acute lung injury.

## Introduction

Exposure to supraphysiological concentrations of oxygen (hyperoxia) leads to lung injury both *in vivo* and *in vitro*. Human patients are exposed to hyperoxia when supplemental oxygen is used for the treatment of critically ill patients with diseases such as acute respiratory distress syndrome (ARDS). Acute respiratory failure due to ARDS has a high mortality^1, 2^. The molecular mechanisms behind acute lung injury (ALI) in this disease process are not well understood. Identification of mechanisms that lead to ALI/ARDS is necessary for the development of new preventive and therapeutic strategies. Hyperoxia is known to contribute to acute lung injury through many molecular mechanisms linked to increased oxidative stress and production of reactive oxygen species^3, 4^. This causes damage to many cellular components such as DNA, lipid and protein, ultimately leading to cell death^5^.

The cytochrome P450 (CYP) family of enzymes are involved in the metabolism of various exogenous and endogenous compounds^6, 7^. Among them, the *CYP1* gene subfamily, regulated by aryl hydrocarbon receptor, is perhaps most relevant to oxygen toxicity^8–13^. Ours and other groups have investigated the role of the CYP1A subfamily in hyperoxic lung injury. CYP1A1 can be induced by hyperoxia and other specific inducers such as 3-methylchloranthene in liver and many other organs including the lung^14^, while CYP1A2 is predominantly expressed in the liver, and is not induced in extra-hepatic tissues even after treatment with inducers. Induction of CYP1A function is protective while inhibition is deleterious in acute hyperoxic lung injury^12, 15^. We have shown that absence of either *Cyp1a1* or *Cyp1a2* gene in knock-out mice increases the susceptibility to hyperoxic lung injury compared to the respective WT controls^16, 17^. The *Cyp1a1*−/− and *Cyp1a2*−/− mice showed greater histological lung injury, inflammation and markers of oxidative stress, hence indicating that both of these proteins are protective in the setting of hyperoxic lung injury. We have previously documented the protective effects of mammalian hepatic CYP1A2 against hyperoxic lung injury^17^, revealing a critical role for extra-pulmonary organs such as liver in the protection against lung injury by metabolizing potential mediators of ALI including lipid hydroperoxides.

Although we have reported on the phenotype of increased lung injury in mice lacking the gene for *Cyp1a1* or *Cyp1a2* and the possible underlying mechanism(s)^16, 17^, the differences in the transcriptome and the underlying biological pathways and regulatory networks have not been investigated. The aims of this study were to identify significant differences in the lung transcriptome and proteome between the wild-type (WT), *Cyp1a1*−/− and *Cyp1a2*−/− mice in a model of acute hyperoxic lung injury. In this study, we employed a non-biased approach to measure global changes in gene expression in WT, *Cyp1a1*−/− and *Cyp1a2*−/− mice following hyperoxia exposure in the lung at 48 h using microarray. We selected the 48 h time point, because most adult mice exposed to hyperoxia survive for 60–90 h with profound lung injury at 72 h of exposure^18^. Instead of focusing on the later cellular injury phase, we wanted to focus on the initiation phase of hyperoxic lung injury^19^. We also sought to measure the changes in the lung proteome using reverse phase protein array (RPPA). Furthermore, because hyperoxia is known to cause oxidative DNA damage^5, 20, 21^ we determined the levels of pulmonary oxidative DNA adducts in WT, *Cyp1a1*−/−, and *Cyp1a2*−/− mice under normoxic as well as hyperoxic conditions.

## Results

### Role of CYP1A in differential pulmonary gene expression in hyperoxic lung injury

**Figure 1:**
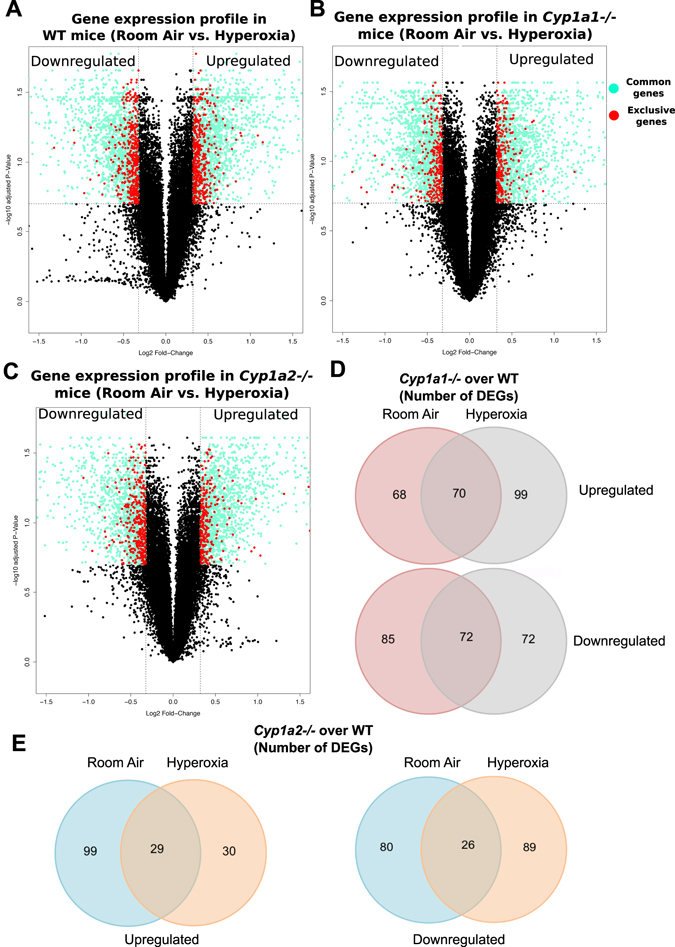
Hyperoxia exposure of WT, *Cyp1a1*−/−, and *Cyp1a2*−/− mice leads to robust yet distinct transcriptomic responses. (**A–C**) Volcano plots of the differentially expressed genes (DEGs); light green: common DEGs across the three genotypes; red: DEGs exclusive to a genotype. (**D**) Number of upregulated (UR) and downregulated (DR) DEGs in *Cyp1a1*−/− and *Cyp1a2*−/− mice compared to WT mice under room air and after hyperoxia exposure. One hundred and fifty three genes (UR:68, DR:85) specific to room air and 171 genes (UR:99, DR:72) specific to hyperoxia were differentially expressed in *Cyp1a1*−/− mice. (**E**) In *Cyp1a2*−/− mice, 179 genes (UR:99, DR:80) specific to room air and 119 genes (UR:30, DR:89) specific to hyperoxia were differentially regulated in the lung compared to WT mice.

To analyze differences in the pulmonary transcriptome after hyperoxia exposure (>95% O_2_ for 48 hrs) we subjected lung mRNA from exposed and room air controls to microarray analysis. Figure 1A–C shows the volcano plots of the differentially expressed genes (DEGs) (upregulated and downregulated) in WT, *Cyp1a1*−/− and *Cyp1a2*−/− mice. The genes shaded in green are common DEGs across the three genotypes whereas the genes represented in red are DEGs exclusive to a genotype. Figures 1D and E show the number of upregulated (UR) and downregulated (DR) DEGs in *Cyp1a1*−/− and *Cyp1a2*−/− mice compared to WT mice under room air and after hyperoxia exposure. One hundred fifty three genes (UR:68, DR:85) in room air and 171 genes (UR:99, DR:72) after hyperoxia exposure were differentially expressed in *Cyp1a1*−/− mice. In *Cyp1a2*−/− mice, 179 genes (UR:99, DR:80) in room air and 119 genes (UR:30, DR:89) specific after hyperoxia exposure were differentially regulated in the lung compared to WT mice. Table 1 shows the exclusive genes (not shared with the other genotypes) with the highest fold change in WT, *Cyp1a1*−/− and *Cyp1a2*−/− mice. Table 2 shows the genes that were downregulated at 48 hrs of hyperoxia exposure in the lung exclusive to a given genotype. The top upregulated and downregulated genes based on fold change, irrespective of the genotype, are shown in supplemental data in Tables S1 and S2. We also determined the genes that were differentially expressed between WT and *Cyp1a1*−/− *or Cyp1a2*−/− mice in room air conditions. The genes showing the highest fold change are listed in Tables S3 (upregulated) and S4 (downregulated). One of the listed genes KLF2 is upregulated in *Cyp1a1*−/− mice and downregulated in *Cyp1a2*−/− mice compared to WT mice in room air conditions. KLF2 has been identified as an important upstream regulator of the lung transcriptome in a model of neonatal hyperoxic lung injury^22^. It is important for lung development in the saccular and alveolar phases^23^. It is also known to be involved in endothelial homeostasis^24^, vascular barrier function^25^ and regulation of inflammation^26^. Baseline differences like above could modulate lung injury upon subsequent exposure to hyperoxia.

**Table 1:** Up regulated genes exclusive to a given genotype (Hyperoxia exposed mice compared to room air controls).

WT		
Gene Symbol	Gene name	Fold change
CD93	Cluster of Differentiation 93	2.21
COL18A1	Collagen Type XVIII Alpha 1	2.13
PPP1R2	Protein Phosphatase 1 Regulatory Inhibitor Subunit 2	1.86
HK1	Hexokinase-1	1.86
PPAP2A	Phosphatidic acid phosphatase 2a	1.84
FKBP1A	FK506 Binding Protein 1A	1.78
NME7	NME/NM23 Family Member 7	1.78
HSP90AA1	Heat Shock Protein 90 kDa Alpha Family Class A Member 1	1.73
ITGA5	Integrin Subunit Alpha 5	1.72
***Cyp1a1*** **−**/**−**		
FAM107A	Family With Sequence Similarity 107 Member A	2.36
SCGB3A1	Secretoglobin, Family 3A, Member 1	1.83
IER3	Immediate Early Response 3	1.69
ALDH1A3	Aldehyde Dehydrogenase 1 Family, Member A3	1.67
HSPB7	Heat Shock Protein Family B Member 7	1.65
LIMS2	LIM Zinc Finger Domain Containing 2	1.60
STT3B	Catalytic Subunit Of The Oligosaccharyltransferase Complex	1.59
CXCL2	Chemokine (C-X-C motif) ligand 2	1.59
CD163	Cluster of Differentiation 163	1.58
***Cyp1a2*** **−**/**−**		
EGR1	Early Growth Response 1	3.77
CYR61	Cysteine-rich angiogenic inducer 61	3.49
ERDR1	Erythroid differentiation regulator 1	3.07
SPINK5	Serine protease inhibitor Kazal-type 5	2.48
MT-ND5	Mitochondrially Encoded NADH:Ubiquinone Oxidoreductase Core Subunit 5	2.04
ZFP36	Zinc finger protein 36	1.95
FOS	FBJ Murine Osteosarcoma Viral Oncogene Homolog	1.90
CEBPB	CCAAT/Enhancer Binding Protein Beta	1.75

**Table 2:** Down regulated genes exclusive to a given genotype (Hyperoxia exposed mice compared to room air controls).

WT		
Gene Symbol	Gene name	Fold change
DOCK6	Dedicator Of Cytokinesis 6	0.60
INMT	Indolethylamine N-Methyltransferase	0.59
IFITM6	Interferon induced transmembrane protein 6	0.58
ATP2A2	ATPase Sarcoplasmic/Endoplasmic Reticulum Ca2+ Transporting 2	0.57
BEX2	Brain Expressed X-Linked 2	0.54
ACTC1	Actin, Alpha, Cardiac Muscle 1	0.54
SDPR	Serum Deprivation Response	0.51
NPPA	Natriuretic Peptide A	0.47
MYL7	Myosin Light Chain 7	0.40
***Cyp1a1*** **−**/**−**		
SERPINA1D	Serpin Family E Member 1	0.56
TCAP	Titin-Cap	0.54
APOA2	Apolipoprotein A2	0.53
SERPINA1B	Serpin Family A Member 1	0.53
IFI27	Interferon Alpha Inducible Protein 27	0.53
MB	Myoglobin	0.49
MUP5	Major urinary protein 5	0.43
SLN	Sarcolipin	0.41
TTR	Transthyretin	0.38
***Cyp1a2*** **−**/**−**		
ALDH1A1	Aldehyde Dehydrogenase 1 Family, Member A1	0.59
OGN	Osteoglycin	0.59
HPGD	Hydroxyprostaglandin Dehydrogenase 15-(NAD)	0.58
ALAS2	5′-Aminolevulinate Synthase 2	0.58
XLR4A	X-linked lymphocyte-regulated 4A	0.57
TAX1BP1	Tax1 Binding Protein 1	0.57
MT-ND4L	Mitochondrially Encoded NADH Dehydrogenase 4L	0.55
COL3A1	Collagen Type III Alpha 1	0.48

### Pathway analysis of DEGs

**Figure 2:**
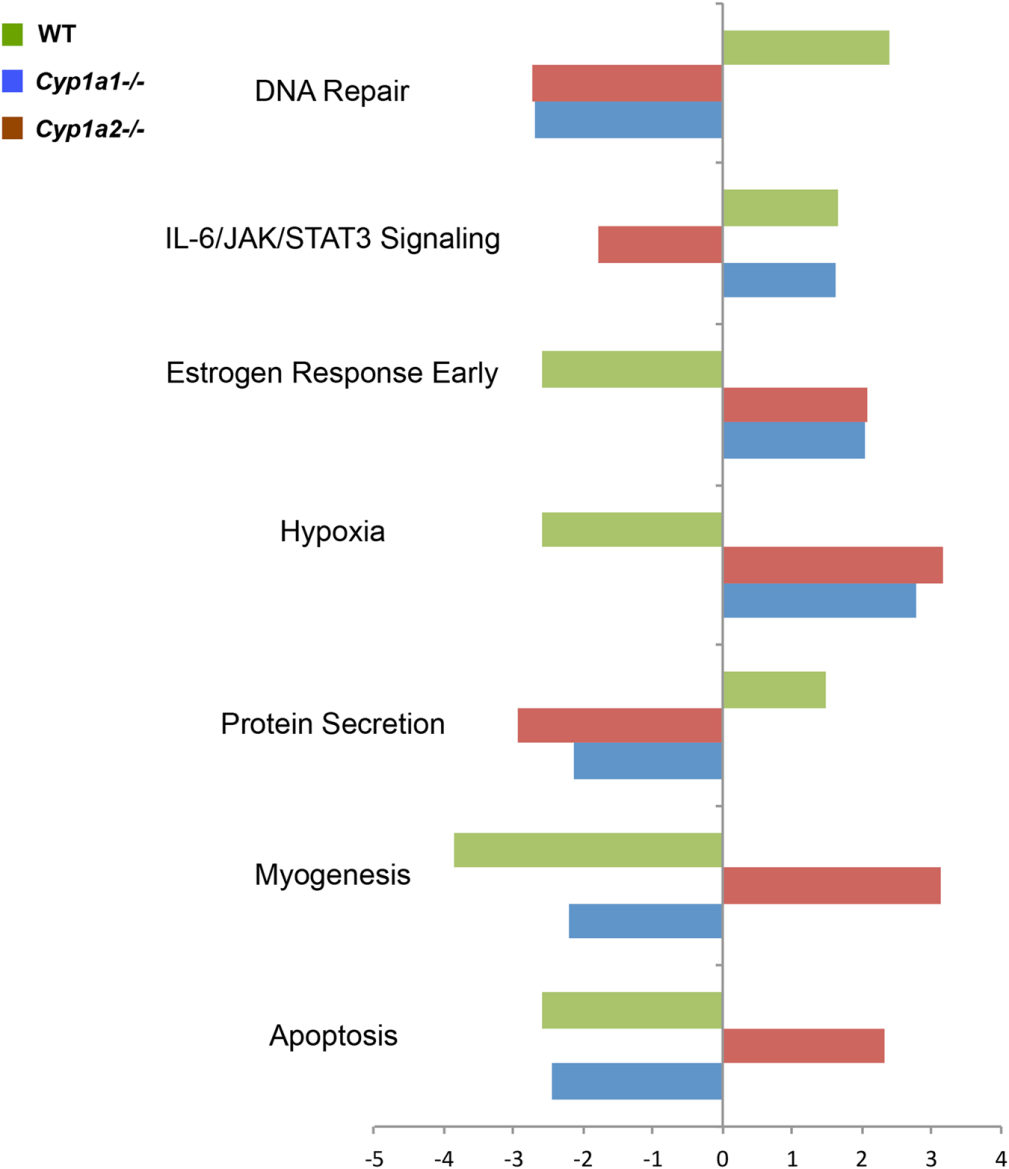
Gene Set Enrichment Analysis (GSEA) reveals distinct modulation of pathways between WT, *Cyp1a1*−/−, and *Cyp1a2*−/−, and WT mice exposed to hyperoxia. Biological processes enriched in the transcriptome footprint of hyperoxia response of three (WT, *Cyp1a1*−/− and *Cyp1a2*−/−) genotypes were identified using Gene Set Enrichment Analysis (GSEA). The Normalized Enrichment Score (NES) is reported for select enriched pathways (fdr-adjusted Q-value < 0.25). An extensive search was carried out for pathways that were enriched after hyperoxia exposure (Q < 0.25) but in driven in opposite direction in WT mice compared to either *Cyp1a1*−/− or *Cyp1a2*−/− mice (NES are significant but have opposite signs for the hyperoxia response in either *Cyp1a1*−/− or *Cyp1a2*−/− compared to the response in WT mice). Key differences were observed in DNA repair and protein secretion (upregulated in WT, downregulated in knock-out) and early estrogen response and hypoxia (downregulated in WT and upregulated in knock-out mice).

Biological processes that were enriched but differentially modulated in the transcriptome footprint of the three (WT, *Cyp1a1*−/− and *Cyp1a2*−/−) genotypes were identified using Gene Set Enrichment Analysis (GSEA). We focused on pathways that were regulated in opposite directions between the WT and *Cyp1a1*−/− or *Cyp1a2*−/− mice. Figure 2 shows the major biological processes for the group of genes that were differentially regulated both in WT, *Cyp1a1*−/− or *Cyp1a2*−/− mice (Q < 0.25; Normalized Enrichment Score/NES has opposed signs between either *Cyp1a1*−/− or *Cyp1a2*−/− and WT). Pathways that were enriched in opposite direction in WT mice compared to *Cyp1a1*−/− and *Cyp1a2*−/− mice were DNA repair and protein secretion (upregulated in WT, downregulated in knock-out) and early estrogen response and hypoxia (downregulated in WT and upregulated in knock-out mice). Among pathways that were differential in *Cyp1a2*−/− mice compared to WT and *Cyp1a1*−/− were IL-6/JAK/STAT3 signaling which were downregulated and apoptosis and myogenesis, which were upregulated in *Cyp1a2*−/− mice after 48 hours of hyperoxia exposure.

### Upstream transcriptional factors analysis

**Figure 3:**
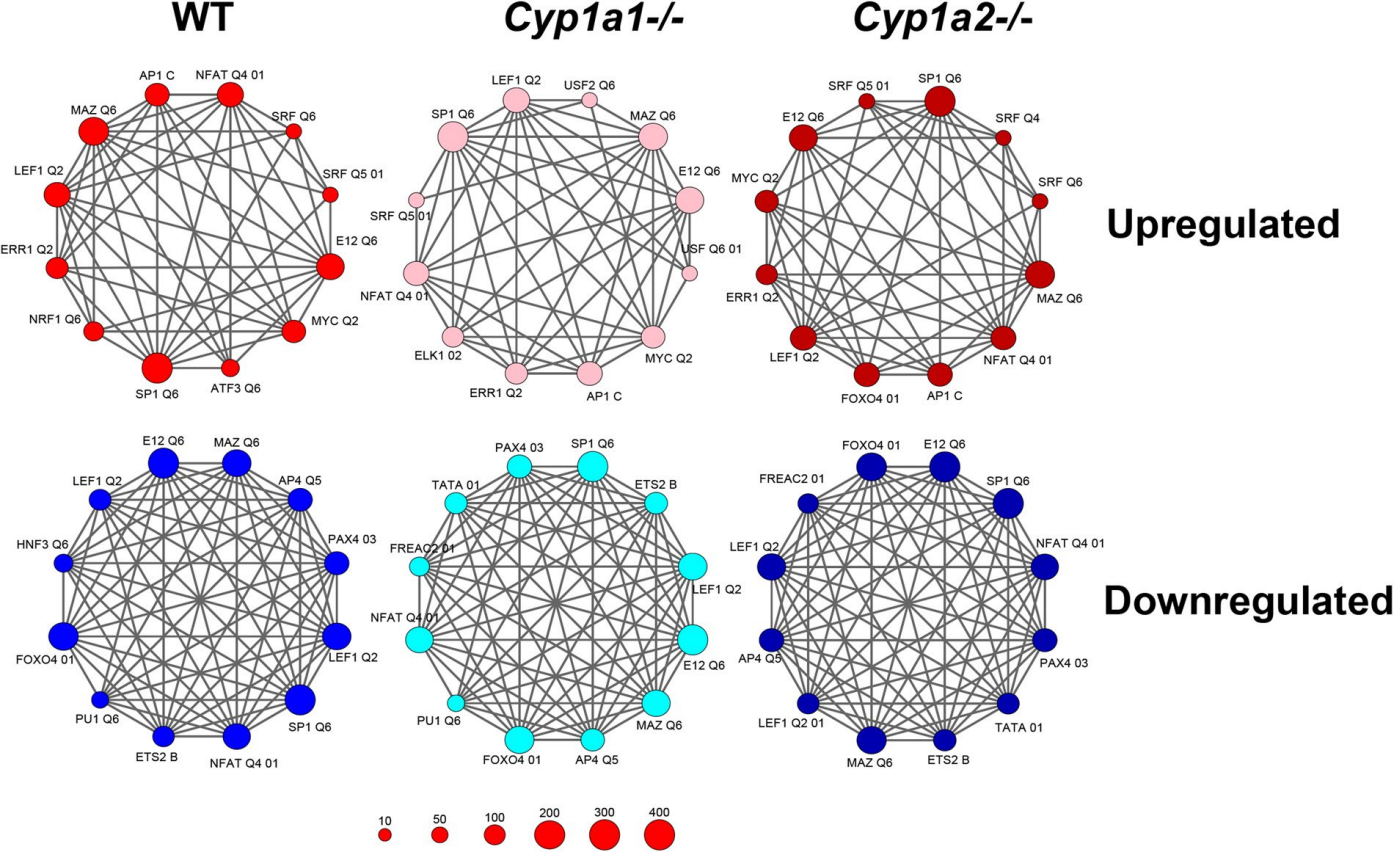
A common network of transcriptional regulators modulates the hyperoxia response. Analysis of upstream transcriptional factors was performed using over-representation analysis (ORA) to identify the key transcription factors modulating the hyperoxia transcriptomic response (hypergeometric distribution; p < 0.05). The top 12 transcriptional regulators in up- and down-regulated genes are depicted as network nodes; edges indicate common gene targets between transcription factors.

**Figure 4:**
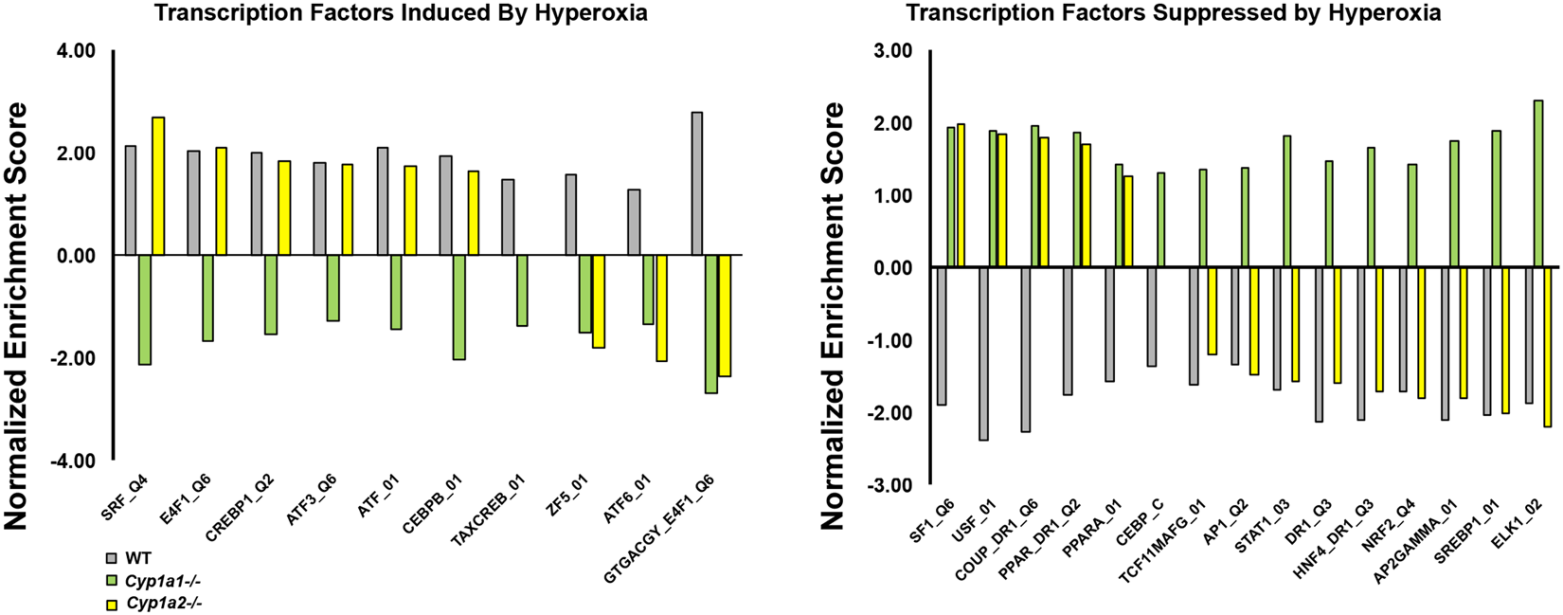
Gene Set Enrichment Analysis (GSEA) reveals distinct modulation of transcriptional regulators between WT, *Cyp1a1*−/−, and *Cyp1a2*−/− mice exposed to hyperoxia. Enrichment of transcriptional regulators in the transcriptomic response of three (WT, *Cyp1a1*−/− and *Cyp1a2*−/−) genotypes after exposure to hyperoxia was assessed using GSEA. An extensive search was carried out for transcriptional regulators that were enriched after hyperoxia exposure (Q < 0.25) but with targets changed in opposite direction in WT mice compared to either *Cyp1a1*−/− or *Cyp1a2*−/− (NES has opposite signs for hyperoxia response in either *Cyp1a1*−/− or *Cyp1a2*−/− compared to the hyperoxia WT mice). We report separately transcriptional regulators with a positive NES (acting primarily as transcriptional activators for WT mice hyperoxia) and those with a negative NES (acting primarily as transcriptional repressors for WT mice hyperoxia).

Next, we sought to analyze the transcription factors (TF) that were responsible for modulating the gene expression changes under hyperoxic conditions in the lung in WT, *Cyp1a1*−/− and *Cyp1a2*−/− mice. The top transcription factors driving the expression of upregulated and downregulated genes in WT, *Cyp1a1*−/− and *Cyp1a2*−/− mice are shown in Fig. 3. Transcription factors which were preferentially induced or suppressed by hyperoxia and were differentially modulated between WT, *Cyp1a1*−/− and *Cyp1a2*−/− mice were determined via GSEA and shown in Fig. 4. Apart from few transcription factors that were regulated in opposite directions between WT and *Cyp1a1*−/− or *Cyp1a2*−/− mice (ZF5, ATF6: induced in WT and PPARA, SF1 and USF suppressed in hyperoxia in WT), the changes were similar between WT and *Cyp1a2*−/− and opposite to those in *Cyp1a1*−/− mice.

### Reverse phase protein array (RPPA)

**Figure 5:**
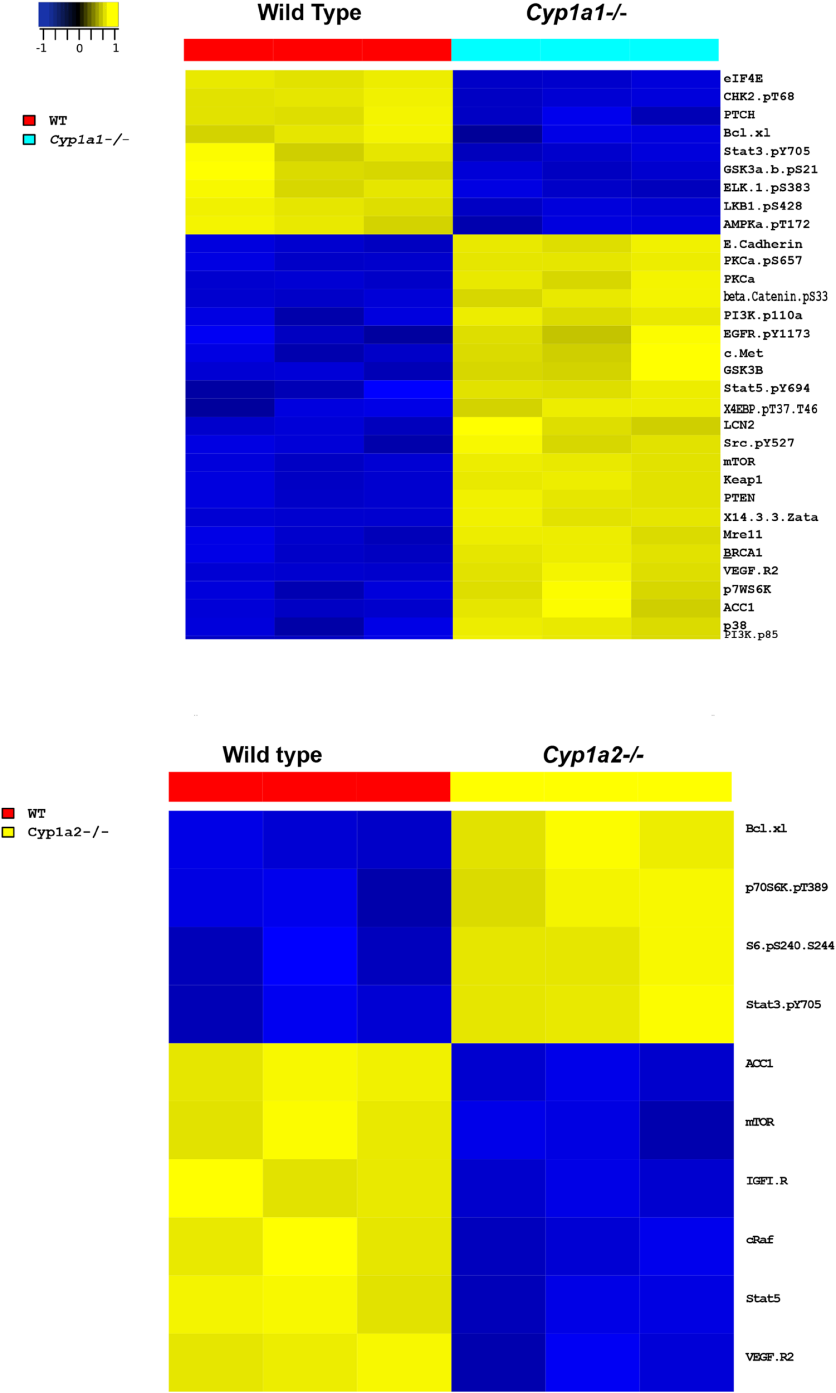
Hyperoxia-exposed *Cyp1a1*−/− and *Cyp1a2*−/− mice are characterized by robust and distinct proteomic changes compared to hyperoxia-exposed WT. Reverse Phase Protein Analysis (RPPA) was used to generate proteomic profiles of WT, *Cyp1a1*−/− and *Cyp1a2*−/− mice exposed to hyperoxia. Differential protein expression was evaluated using parametric t-test (significance for fdr-adjusted q-value < 0.25). For a uniform visual representation, expression of each protein were z-score transformed; e.g. for each protein, values were first mean-centered across all samples, then further divided by the standard deviation across all samples. A. Proteomic changes of *Cyp1a1*−/− mice compared to WT mice exposed to hyperoxia B. Proteomic changes of *Cyp1a2*−/− mice compared to WT mice exposed to hyperoxia.

For analysis of the lung proteome we performed RPPA analysis on lung protein samples after 48 hours of hyperoxia exposure and respective room air controls. Figure 5 shows the differences between WT and *Cyp1a* knockout animals under hyperoxic conditions. We did not see significant differences in protein expression in the *Cyp1a2*−/− mice exposed to hyperoxia compared to their room air controls. Supplemental Figure 1 shows the differences between hyperoxia exposed and room air controls in each respective genotype. Among the proteins studied, in WT mice, 27 were downregulated and 8 were upregulated compared to room air controls and in *Cyp1a1*−/− mice, 21 proteins were downregulated and 10 upregulated upon exposure to hyperoxia (Supplemental Figure 1). The protein and corresponding genes differentially expressed between WT and *Cyp1a* knockout animals under hyperoxic conditions are listed in supplemental data Table S5 and those between hyperoxia exposed and room air controls in each respective genotype are listed in Table S6. Many of the significantly changed proteins detected by the RPPA assay were post-translationally modified, and as such their expression may not have been assessed as significantly changed in the microarray profile.

### Real time qPCR validation

**Figure 6:**
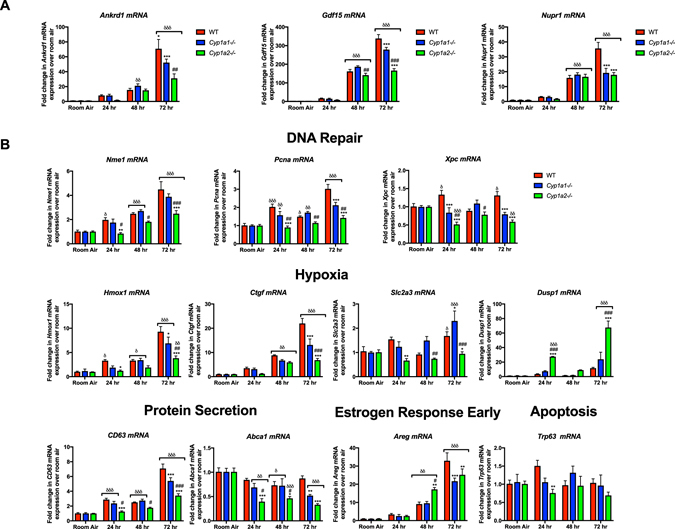
Real time RT-PCR analysis of mRNA from the lungs of WT, *Cyp1a1* and *Cyp1a2*−/− mice exposed to room air or hyperoxia for 24, 48 or 72 h shows differential regulation of gene targets among WT *Cyp1a1* and *Cyp1a2*−/− mice. Fold change over room air levels are represented on the y-axis. Two way ANOVA was used to assess statistical significance in adduct levels among genotypes and the effect of hyperoxia. (**A**) Differences in expression of *Ankrd1*, *Gdf15* and *Nupr1*; genes identified to be upregulated among all three genotypes upon exposure to hyperoxia. (**B**) Differential expression of genes within pathways that were differentially modulated between WT and *Cyp1a1*−/− or *Cyp1a2*−/− mice. Significant differences in expression compared to levels in WT mice are represented by *p < 0.05, **p < 0.01 and ***p < 0.001. Significant differences between *Cyp1a1*−/− and *Cyp1a2*−/− mice are represented by ^#^p < 0.05, ^##^p < 0.01 and ^###^p < 0.001. Significant differences from room air levels are represented by ^δ^p < 0.05, ^δδ^p < 0.01 and ^δδδ^p < 0.001.

Microarray results were verified by qPCR. Instead of choosing targets based solely on fold change for validation, we selected candidate genes from the pathways that were differentially regulated between *WT*, *Cyp1a1*−/− *and Cyp1a2*−/− *mice*. These included (*Nme1*, *Pcna and Xpc*: DNA repair pathway; *Hmox1*, *Ctgf*, *Slc2a3*, *Dusp1*: response to hypoxia pathway; *Cd63*, *Abca1*: protein secretion pathway; *Areg* from early estrogen response pathway and *Trp63* from the apoptosis pathway). This was based on the key insight that small but consistent changes across a large number of genes in a pathway (including genes that do not meet a certain statistical threshold) are more informative than large significant changes in a handful of genes only^27^. We assessed differences in pulmonary gene expression at 24 and 72 h in addition to 48 h to observe the temporal changes related to duration of hyperoxia exposure (Fig. 6). The fold change for each gene was calculated relative to its expression levels in the respective room air controls. We also validated three genes which were commonly upregulated in all three genotypes: *Ankrd1*, *Gdf15 and Nupr1*.

The DNA repair pathway genes; *Nme1*, *and Pcna* were induced by hyperoxia in WT and *Cyp1a1*−/− mice compared to room air levels; increasing duration of hyperoxia exposure led to greater fold increases. Interestingly, in *Cyp1a2*−/− mice, there was decreased expression of these genes compared to WT and *Cyp1a1*−/− mice. Expression of *Xpc* was increased in WT mice at 24 and 72 h compared to room air controls but *Cyp1a1*−/− *and Cyp1a2*−/− mice showed decreased expression levels compared to WT mice under hyperoxic conditions. *Cyp1a2*−/− mice exhibited consistently the lowest expression for these genes among the three genotypes.

Among genes involved in response to hypoxia: *Hmox1* and *Ctgf* were induced by hyperoxia with increasing expression with increasing duration of hyperoxia exposure. The induction was very significant in WT mice and to a lesser extent in *Cyp1a1*−/− mice, and the least induction was observed in *Cyp1a2*−/− mice. *Slc2a3* was induced at 72 h in WT and *Cyp1a1*−/− mice compared to room air controls but decreased expression was seen in *Cyp1a2*−/− mice compared to other genotypes. However, expression of *Dusp1* was significantly increased in *Cyp1a2*−/− mice at 24 and 72 h compared to other genotypes and respective room air controls.

We validated *Cd63* and *Abca1* as part of the protein secretion pathway. *Cd63* was induced under hyperoxia in all three genotypes but was most significant in WT mice and the least in *Cyp1a2*−/− mice. On the other hand, expression levels of *Abca1* decreased under hyperoxic conditions in all three genotypes but this was more significant in the *Cyp1a1*−/− *and Cyp1a2*−/− mice compared to WT mice at 72 h. *Areg* (early response to Estrogen pathway) expression was increased at 48 and 72 h in all three genotypes after hyperoxia exposure at 48 and 72 h. Expression was higher in *Cyp1a2*−/− mice at 48 h but at 72 h, WT mice had higher expression levels compared to *Cyp1a1*−/− *and Cyp1a2*−/− mice. *Trp63* (Apoptosis pathway) did not show significant change in expression with hyperoxia. The expression was decreased in *Cyp1a2*−/− mice at 24 h compared to WT mice.

Expression levels of *Ankrd1*, *Gdf15 and Nupr1* were significantly increased in all three genotypes at 48 and 72 h compared to room air controls. At 72 h, the expression was lower in *Cyp1a1*−/− and *Cyp1a2*−/− mice compared to similarly exposed WT mice.

There were excellent correlations in fold changes measured by real time PCR and by microarray for the experimentally validated genes, as shown in Table 3. The Pearson Correlation Coefficient for fold change in gene expression was 0.85 for WT, 0.84 for *Cyp1a1*−/− and 0.78 for *Cyp1a2*

**Table 3:** Table showing Fold change in gene expression for selected genes with Real time PCR and Microarray.

Gene Name	Genes	PCR (Fold change from room air)			Microarray (Fold change from room air)		
Growth differentiation factor 15	GDF15	161.6	186.5	141.4	19.85	18.19	18.49
Ankyrin repeat domain 1	ANKRD1	17.63	21.07	13.52	6.49	10.22	8.63
Nuclear protein 1, transcriptional regulator	NUPR1	14.25	18.12	16.56	13.41	11.31	15.19
CD63 molecule	CD63	1.82	2.23	1.85	1.96	2.01	1.74
ATP binding cassette subfamily A member 1	ABCA1	0.8	0.61	0.44	0.84	0.82	0.74
XPC complex subunit, DNA damage recognition and repair factor	XPC	0.92	1.17	0.73	0.86	0.89	0.77
NME/NM23 nucleoside diphosphate kinase 1	NME1	2.48	2.71	1.82	1.92	1.96	1.73
Proliferating cell nuclear antigen	PCNA	1.49	1.72	1.19	1.04	1.00	0.92
Solute carrier family 2 member 3	SLC2A3	0.92	1.49	0.74	0.91	1.13	0.83
Tumor Protein P63	TRP63	0.97	1.32	0.7	0.95	1.03	1.00
Amphiregulin	AREG	8.04	9.54	17.17	3.90	4.36	7.72
Connective tissue growth factor	CTGF	8.81	6.71	5.94	5.47	3.33	5.04
Heme oxygenase 1	HMOX1	3.54	3.43	2.25	1.60	1.99	1.48
Dual specificity phosphatase 1	DUSP1	1.68	1.76	9.08	0.79	0.91	2.00

−/− mice.

### Immunoblot analysis

**Figure 7:**
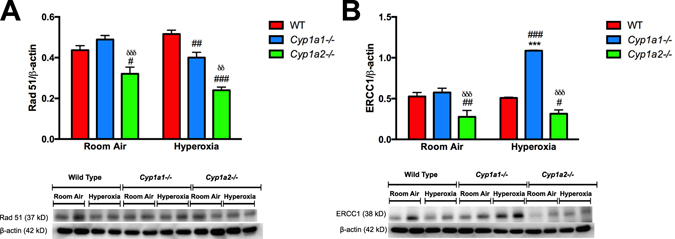
Effect of hyperoxia on Rad 51 (**A**) and ERCC1 (**B**) protein expression in WT, *Cyp1a1*−/− and *Cyp1a2*−/− mice in room air and after 48 h of hyperoxia. For western blotting, lung whole protein (20 μg of protein) from individual animals (n = 4/group) exposed to room air or to hyperoxia for 48 h. For loading controls, the membranes were stripped and incubated with antibodies against β-actin, followed by electrochemical detection of bands. Two way ANOVA was used to assess statistical significance in protein expression among genotypes and the effect of hyperoxia. Significant differences from room air controls are represented by **p < 0.01 and ***p < 0.001. Significant differences in expression compared to levels in WT mice is represented by ^#^p < 0.05, ^##^p < 0.01 and ^###^p < 0.001. Significant differences in expression compared to levels in *Cyp1a1*−/− mice is represented by ^δ^p < 0.05, ^δδ^p < 0.01 and ^δδδ^p < 0.001.

For validation at the protein level we performed western blots for Rad51 and ERCC1. These genes are both part of the DNA repair pathway and were hence chosen for further validation at the protein level. These results are shown in Fig. 7. Protein expression assessed by western blot assay in whole lung protein from WT, *Cyp1a1*−/− *and Cyp1a2*−/− mice exposed to room air and after 48 h hyperoxia exposure was measured and quantified using densitometry. *Cyp1a2*−/− mice had decreased expression of Rad51 and ERCC1 compared to WT or *Cyp1a1*−/− mice both under room air and after exposure to hyperoxia. *Cyp1a1*−/− mice had decreased expression of Rad 51 and a significantly increased expression of ERCC1 after hyperoxia exposure compared to WT mice.

### Lung oxidative DNA adducts analysis in WT, *Cyp1a1*−/− and *Cyp1a2*−/− mice in acute hyperoxic lung injury

**Figure 8:**
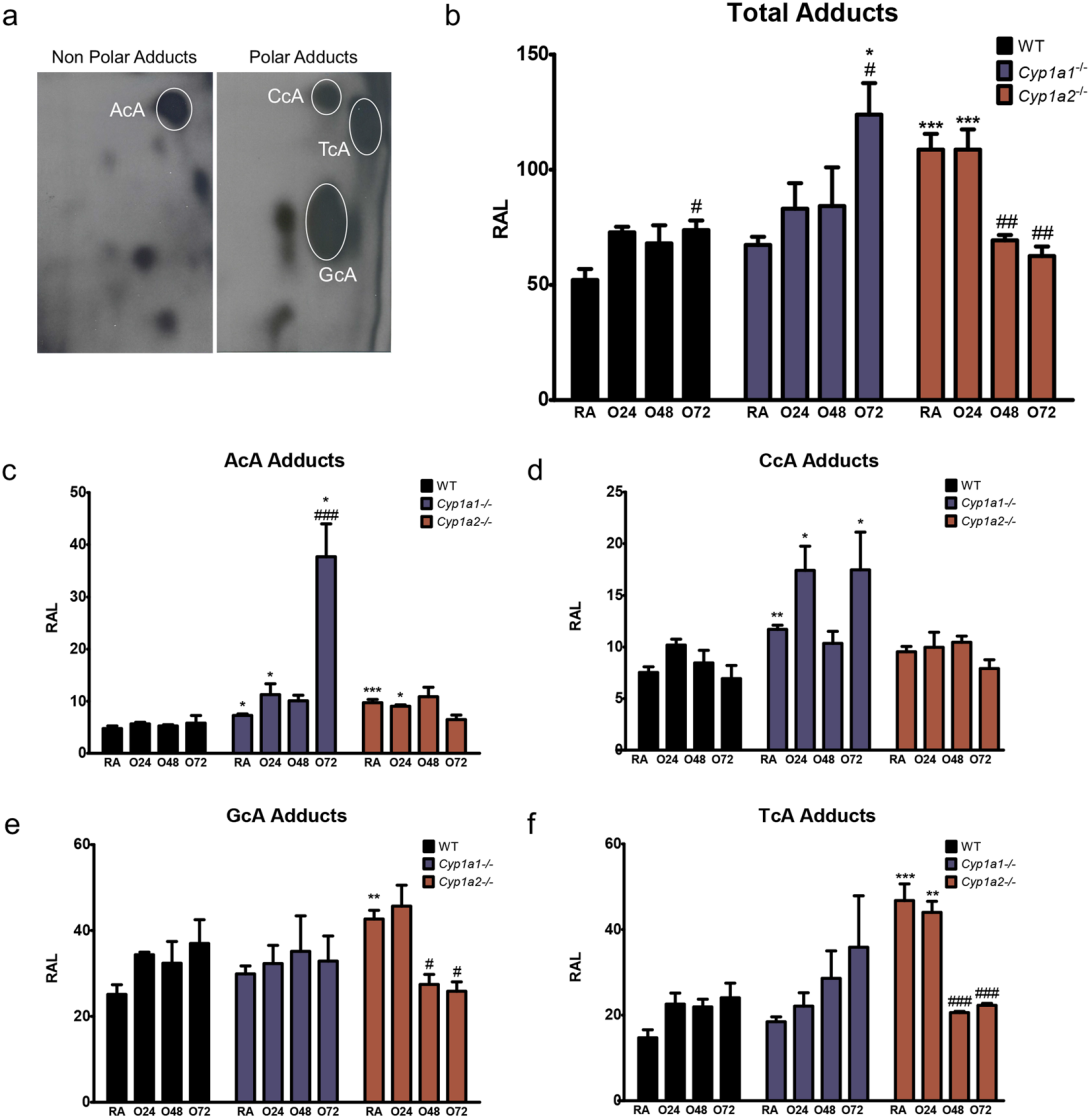
Pulmonary 8,5′-cyclo-2′-deoxyadenosine (cA) oxidative DNA adducts in WT *Cyp1a1*−/−, and *Cyp1a2*−/− mice exposed to hyperoxia. (**a**) Representative thin-layer chromatography maps displaying bulky oxidative DNA adducts. Polar and non-polar oxidative DNA adducts were detected by ^32^P-postlabeling, as described in Materials and Methods. Panels (b–f) show quantitative analyses of total cA, AcA, CcA, GcA, and TcA adducts in the lung in WT, *Cyp1a1* ^−/−^, and *Cyp1a2* ^−/−^ mice (n = 3/group) that were maintained in room air (RA) or exposed to 24, 48, and 72 hours of hyperoxia. The total cA values were derived from the addition of the individual adduct values. Two way ANOVA was used to assess statistical significance in adduct levels among genotypes and the effect of hyperoxia. Significant differences in adduct levels between WT and *Cyp1a1* ^−/−^ or *Cyp1a2* ^−/−^ mice is represented by *p < 0.05, **p < 0.01 and ***p < 0.001. Significant differences between normoxic (RA) and hyperoxic animals are represented by ^#^p < 0.05, ^##^p < 0.01, and ^###^p < 0.001.

Oxidative DNA adducts were analyzed in the lungs by ^32^P-postlabeling. The 8,5′-Cyclo-2′-deoxyadenosine (cA) adducts that we measured in this study represent one of the major oxidative DNA lesions formed in DNA by hydroxyl radical attack on 2′-deoxyadenosine, followed by intramolecular cyclization between C5 and C8^28, 29^. Specifically, we measured levels of the dinucleotides AcA, GcA, CcA, and TcA, since they have been proposed as novel biomarkers of oxidative DNA damage, and were robustly increased under conditions of oxidative stress *in vivo and in vitro* ^30, 31^. Figure 8(A) shows the typical profile of non-polar (AcA) and polar (GcA, CcA, and TcA) oxidative DNA lesions. In WT mice and *Cyp1a1*−/− mice, the total adducts were significantly augmented after 72 h of hyperoxia (Fig. 8b) and the levels were higher in *Cyp1a1*−/− mice compared to WT mice. On the other hand, the *Cyp1a2*−/− mice showed increased total adduct levels at room air and 24 h compared to similarly exposed WT mice, and the total adduct levels in the *Cyp1a2*−/− mice decreased at the 48 and 72 h time points (Fig. 8b).

In regard to individual adducts, WT mice did not exhibit significant hanges after hyperoxia exposure (Fig. 8c–f). In *Cyp1a1*−/− mice, the AcA adducts were significantly higher at 72 compared to room air. Upon comparison with WT mice, *Cyp1a1*−/− mice had higher levels of AcA and CcA adducts at room air and after 24 and 72 h of hyperoxia exposure (Fig. 8c,d). GcA and TcA adduct levels were higher in *Cyp1a2*−/− mice at room air and 24 h compared to WT mice. The GcA and TcA adducts were markedly attenuated after 48–72 h of hyperoxia in *Cyp1a2*−/− mice. Some additional spots were formed in the DNA of mice both in room air and hyperoxic conditions. We do not know the identity of these modifications, and we will characterize these compounds in future studies.

## Discussion

The key findings of this study were the identification of differentially regulated molecular pathways in hyperoxic lung injury that may explain the susceptibility of *Cyp1a1*−/− and *Cyp1a2*−/− mice to acute lung injury in this model. Differences in the lung transcriptome have been described in hyperoxic lung injury^32–34^ but the role of (CYP)1A enzymes in modulating the lung transcriptome under hyperoxic conditions using knock-out animal models has not been previously reported. We have shown previously that hepatic CYP1A2 protects against hyperoxic lung injury by decreasing lipid peroxidation and oxidative stress *in vivo* ^17^, *thus* revealing a critical protective role for extra-pulmonary organs such as liver. F_2_-isoprostanes/isofurans that are formed during hyperoxia exposure may undergo detoxification by CYP1A2 to non-toxic metabolites. Through *in vitro* experiments with recombinant human CYP1A2, we demonstrated the formation of a dinor metabolite from PGF2-α showing that F_2_-isoprostanes are endogenous substrates for CYP1A2^17^. In *Cyp1a2*−/− mice, these compounds would accumulate, be transported to the systemic circulation, and lead to increased lung injury. Using a similar methodology, we also showed that CYP1A1 has a protective role in part due to CYP1A1-mediated decrease in the levels of reactive oxygen species-mediated lipid hydroperoxides *e*.*g*. F_2_-isoprostanes/isofurans, leading to attenuation of oxidative damage^16^. Compounds such as omeprazole, a proton pump inhibitor and beta-naphtoflavone, a flavonoid have been shown to attenuate hyperoxic lung injury by inducing CYP1A^12, 13, 35^.

We reported differential gene expression in the lungs after hyperoxia exposure for 48 hours and identified genes that are differentially expressed unique to each of the genotypes as well as those that are common. We analyzed differences in gene expression at the 48 h time point because most animals displayed severe lung injury by 72 h, and many died between 60–90 h of exposure^34^. We focused on pathways that were regulated in opposite directions between the WT and *Cyp1a1*−/− or *Cyp1a2*−/− mice. DNA repair and protein secretion pathways were upregulated in WT and downregulated in knock-out, whereas the reverse was the case for early estrogen response and hypoxia pathways.

### DNA Repair Pathway

Reactive oxygen species (ROS) generated during hyperoxic conditions can cause damage to many cellular components including DNA, which has been reported to contribute to the development of diseases such as cancer and cardiopulmonary diseases including ARDS^4, 36^. DNA damage and induction of DNA repair genes has been reported in the lung following response to hyperoxia both *in vivo* and *in vitro* ^37, 38^. Upregulation of the DNA repair pathway is critical for the damaged cells to stimulate repair and prevent the proliferation of cells with DNA damage. Cell cycle arrest at checkpoints is one such defense mechanism, which prevents the introduction of deleterious mutations. In WT mice, we observed upregulation of this pathway in pathway analysis and increased expression of genes such as *Nme1* and *Pcna* at the mRNA level. NME1 (Nucleoside Diphosphate Kinase 1) is a 3′–5′ exonuclease that participates in DNA repair. In human cells, its expression increases following DNA damage^39, 40^. PCNA (proliferating cell nuclear antigen) plays a key role in DNA damage response by coordinating DNA replication with DNA repair^41^. This pathway was downregulated in the knock-out animals under pathway analysis, which could explain the diminished DNA repair and increased cell death, and in turn it might lead to increased lung injury in these animals as, described before^16, 17^. *Cyp1a2*−/− mice showed significantly decreased expression of genes (at the mRNA and the protein level) in this pathway including *Xpc* (Xeroderma Pigmentosum, Complementation Group C), which neutralizes oxidative DNA damage by playing a role in nucleotide excision repair^42, 43^. *Cyp1a1*−/− mice showed decreased protein levels of Rad51 compared to WT mice after exposure to hyperoxia. Rad 51 plays a major role in DNA double-strand break repair and prevents genomic instability and the generation of tumorigenic mutations^44^. However, there was increased expression of ERCC1 protein (Excision Repair Cross-Complementation Group 1) in *Cyp1a1*−/− mice after hyperoxia exposure compared to WT mice and room air controls. This gene is involved in recombinational DNA repair and in the repair of inter-strand crosslinks^45^. Total adducts were significantly increased in *Cyp1a1*−/− mice compared to WT mice and room air controls, hence the increase in ERCC1 could have been in response to the increased adduct formation in these mice.

### Protein Secretion Pathway

The candidate genes in the protein secretion pathway that were further evaluated using PCR were: *Cd63* and *Abca1* (ATP Binding Cassette Subfamily A Member 1). CD63 belongs to the tetraspanin family of cell-surface proteins. They mediate signal transduction events and play a role in the regulation of cell development, activation, growth and motility. *Cd63* was induced under hyperoxia in all three genotypes but was most increased in WT mice and the least in *Cyp1a2*−/− mice. Doyle *et al*. showed that CD63 is essential for leucocyte recruitment by endothelial cells^46^. It could thus play a pro-inflammatory role in this model of acute lung injury. ABCA1 is a member of the ABC1 subfamily. This protein functions as a cholesterol efflux pump in the cellular lipid removal pathway. In LPS induced acute lung injury, this protein was found to be protective and decreased inflammation^47^. It was downregulated in hyperoxia exposed mice in all three genotypes but the decreases were more pronounced in the *Cyp1a1*−/− *and Cyp1a2*−/− mice compared to WT mice at 72 h.

### Response to Hypoxia Pathway

DUSP1 (Dual Specificity Phosphatase 1) protein has intrinsic phosphatase activity, and specifically inactivates mitogen-activated protein (MAP) kinase. DUSP1 expression is increased under conditions of increased oxidative stress and apoptosis^48^. In this study, DUSP1 expression was significantly increased in *Cyp1a2*−/− mice, which may be indicative of increased oxidative stress in these animals. This was consistent with our previous report of increased oxidative stress in the lungs of *Cyp1a2*−/− mice when exposed to hyperoxia compared to WT mice^17^. Hmox1 (Heme oxygenase 1) was up regulated in both WT and *Cyp1a1*−/− and to a significantly lesser extent in *Cyp1a2*−/− mice after hyperoxia exposure. Previous investigators have reported up regulation of this gene in the lung under hyperoxic conditions^49–51^. Both protective and deleterious effects of its induction have been reported. This could be because of cell-specific effects or linked to the byproducts of Hmox1 mediated reaction including free iron or carbon-monoxide^52–55^. *Ctgf* (Connective tissue growth factor) mRNA was decreased in the knock-out mice and increased in the WT mice after hyperoxia exposure in this model. Increase in CTGF expression in WT mice has been reported in hyperoxia models^56, 57^ and in models with bleomycin induced pulmonary fibrosis^58^ where it is thought to have pro-fibrotic effect. *Slc2a3* (or GLUT3: Glucose transporter type 3) is involved in transmembrane transport of glucose and is up regulated in hypoxic conditions possibly though the HIF-1alpha pathway^59^. Expression was increased at 72 h in WT and *Cyp1a1*−/− mice compared to room air controls, but decreased expression was seen in *Cyp1a2*−/− mice compared to other genotypes.

### Early Estrogen Response pathway

The early estrogen pathway was also differentially modulated between the genotypes; downregulated in WT, but upregulated in the knock-out animals. This pathway comprises of genes that define early response to estrogen. *Caldon et al*. proposed that estrogen receptor signaling may compromise effective DNA repair and cellular apoptosis in favor of proliferation^60^. In this study, we showed increased activation of the DNA repair pathway in WT mice but this was less in the knock-out mice corroborating the hypothesis above. We looked into Amphiregulin (*Areg*) expression by PCR. It was significantly increased in the lungs of *Cyp1a2*−/− mice compared to WT mice but all genotypes showed increased expression following hyperoxia exposure. Amphiregulin is known to mediate estrogen signaling^61^. It also increases TGF-β mediated lung fibrosis by functioning as a ligand for EGF receptor^62^. Expression of *Areg* was increased in the newborn lung exposed to chronic hyperoxia^63^ and may have a pro-inflammatory effect in HLI as noted in other diseases^64^.

### Apoptosis pathway

*Trp63* or *p63* is a transcription factor of *p53* gene family involved in cell differentiation and response to stress. It also functions as a master regulator of epidermal development^65^. *Trp63* regulates p53 function and has been shown to have pro-survival function by antagonizing p53 activity^66, 67^. In our study, we found significant down-regulation of this gene in *Cyp1a2*−/− mice indicating its potential role in the increased lung injury phenotype observed in these mice.

Induction in *Ankrd1* and *Gdf15* expression under hyperoxic conditions has been reported in WT mice^34^. Overexpression of *Ankrd1* (Ankyrin repeat domain 1) has been shown to enhance apoptosis^68^ through the p53 pathway. *Gdf15* (Growth Differentiation factor 15) is a member of the TGF-β superfamily and is widely distributed in mammalian tissues. It is a stress-responsive cytokine and we have previously shown that it has a pro-survival and anti-oxidant role in hyperoxia *in vitro* ^69^. *Nupr1* (Nuclear Protein 1, Transcriptional Regulator) is a stress activated protein known to regulate apoptosis, cell cycle and regulation of TGF-β activity^70^. Induction of *Nupr1* by hyperoxia has been reported in the newborn mouse lung exposed to hyperoxia^71^ and in the newborn retina following hyperoxia exposure^72^.

The fact that a significant level of bulky oxidative DNA adducts were formed in room air conditions suggests that even during normoxic conditions, ROS can be produced at levels that result in DNA damage. The increase in total adducts levels after 24–72 h of hyperoxia in WT animals (Fig. 8) was probably due to increased oxidative stress induced by oxygen. The higher levels of GcA and TcA adducts in WT mice suggests that these bases are more susceptible to oxidative attack that adenosine or cytosine. The presence of adducts after as early as 24 h of hyperoxia exposure supports the hypothesis that oxidative DNA adducts mechanistically contribute to lung injury. Our observation that in mice lacking the *Cyp1a1* gene the total adducts were significantly augmented after 72 h of hyperoxia (Fig. 8D) supports the idea that CYP1A1 plays a role in the detoxification of the adduct precursors. Since *Cyp1a1*−/− mice display increased lung injury compared to the WT mice^16^, we postulate that oxidative DNA injury has a causative role in lung injury.

Although the *Cyp1a2*−/− mice were even more susceptible to hyperoxic lung injury than *Cyp1a1*−/− mice^17, 73^, the fact that the *Cyp1a2*−/− mice showed significantly higher levels of total oxidative DNA adducts under room air conditions than those in WT mice (please compare Fig. 8E and C) suggests that CYP1A2 plays an important role in the detoxification of the adduct precursors. As these adducts are may have already reached physiological saturation, higher oxygen levels as seen at 24–72 h after hyperoxia may not have resulted in increased oxidative DNA damage in these animals. Interestingly, the levels of oxidative DNA lesions were attenuated in *Cyp1a2*−/− mice after prolonged hyperoxia (Fig. 8b–f), and this could be due to enhanced DNA repair of these specific dinucleotides *in vivo*, but further experimental proof is needed to substantiate this idea.

The differences in lung proteome as analyzed with the RPPA methodology showed that there were greater number of proteins differentially expressed between WT and *Cyp1a1*−/− than WT and *Cyp1a2*−/− mice. These differences may underlie the different lung injury phenotypes observed in these animals. For example: mTOR (mechanistic Target of Rapamycin), was downregulated in both WT and *Cyp1a1*−/− mice compared to room air controls in hyperoxia. Inhibition of the mTOR pathway has been shown to decrease hyperoxia-induced lung injury by increasing autophagy^74^. Similarly expression of VEGFR2 was decreased in both WT and *Cyp1a1*−/− mice. Decreased expression of this gene under hyperoxic conditions has been reported previously^75^. Among the upregulated proteins were phosphorylated ACC1; Acetyl-CoA carboxylase (2.22 fold in WT and 1.22 fold in Cyp1a1−/− mice) that catalyzes the ATP-dependent carboxylation of acetyl-CoA to produce malonyl-CoA, the pivotal step in the fatty acid synthesis pathway. Phosphorylation inhibits the activity of ACC1 and is observed in conditions when the energy status of the cell is low. Protein expression of phosphorylated AMPK (5′-prime-AMP-activated protein kinase) is also increased in both WT and *Cyp1a1*−/− mice, which is the activate form of this protein, and leads to downstream phosphorylation and inhibition of ACC1 as was observed in our study^76, 77^.

We also highlighted the various transcription factors, which could have been differentially regulated between the genotypes in hyperoxic lung injury. TF genes are usually not significantly up- or downregulated in microarray experiments. Their activity is mainly regulated at the level of ligand binding or at the posttranscriptional level. One example is: C/EBPβ (ccat/enhancer binding protein), which was induced in WT and *Cyp1a2*−/− mice, but suppressed in *Cyp1a1*−/− mice. The (C/EBP) family of proteins are transcription factors that respond to extracellular signals and regulate cell proliferation and differentiation^78^. Increased expression of C/EBPβ has been reported in rats exposed to hyperoxia *in vivo* ^79^. Ramsay *et al*. showed that hyperoxia exposure resulted in increased expression of the C/EBP beta isoform: liver-inhibiting protein (LIP), which decreases the transcription of CCSP (Clara cell secretory protein) which is protective under conditions of oxidant lung injury^80^.

In conclusion, we present the changes in the mouse pulmonary transcriptome and proteome after hyperoxia exposure at the 48 h time point in WT, *Cyp1a1*−/− and *Cyp1a2*−/− mice. We identified DEGs involved in apoptosis, DNA repair and estrogen response pathways that may explain the differences in susceptibility of *Cyp1a1*−/− and *Cyp1a2*−/− mice to HLI. We also highlighted the differences in the DNA repair pathway and highlighted involved genes and proteins that could explain the differences in DNA injury observed in the knock-out animals. These findings provide novel insights into the mechanisms involved in HLI and suggest new pathways that need to be investigated as possible preventative and/or therapeutic targets against acute lung injury in humans.

## Methods

### Animals

Approval for this study was obtained from the Institutional Animal Care and Use Committee (IACUC) of Baylor College of Medicine (Protocol number AN-907). All experiments were performed in accordance with relevant guidelines and regulations. Care of animals in research met the highest contemporary standards as per the 8^th^ edition of the guide for the care and use of laboratory animals and other IACUC protocols. C57BL/6J wild type (WT) mice were obtained from Charles River laboratories (Wilmington, DE) and creation of the *Cyp1a1*(*–*/*–*) knockout mouse line, which was on C57BL6 background, has been described before^81^. Creation of the *Cyp1a2*(−/−) knockout mouse line, has been previously described^81^. These mice, which were on a mixed background (B6/SV129) were cross-bred into the C57BL/6J background by back-crossing for 12 generations, resulting in *Cyp1a2*(−/−) on >99% B6 background. Eight to ten week old mice were maintained at Texas Children’s Hospital animal facility and used for the study. They were fed standard mice food and water *ad libitum* and maintained in a 12 h day/night cycle.

### Oxygen exposure

Adult male mice (8–10 week-old) were maintained in either room air (21% oxygen) or exposed to hyperoxia (95–100% oxygen) using pure O_2_ at 5 l/min for 48 hrs in a sealed Plexiglass chamber, as reported previously^8^. We measured the oxygen concentration in the plexiglass chamber by an analyzer (Getronics, Kenilworth, New Jersey). After hyperoxia exposure, the animals were anesthetized with 200 mg/kg of sodium pentobarbital (i.p.) and euthanized by exsanguination while under deep pentobarbital anesthesia. The lung tissues were harvested for further analysis.

### RNA isolation

We used 3 animals per genotype per treatment group. The samples were not pooled. Total RNA from frozen lung samples was isolated using the miRNeasy kit as per the manufacturer’s standard protocols (Qiagen, Valencia, CA, USA). Sample concentration was assayed using a Nanodrop-8000 (Thermo Scientific, Wilmington, DE, USA) and quality checks were done using the NanoDrop spectrophotometer and the Agilent Bioanalyzer. 250 ng of total RNA was reverse transcribed, and microarray hybridization performed using the Illumina Gene Expression MouseWG-6 v2.0 Expression BeadChip Kit at the Laboratory for Translational Genomics at Baylor College of Medicine. The transcriptome profile data was quartile-normalized by the Bioconductor lumi package^82^. RNA quality parameter were as follows: The 260/280 and 260/230 ratios needed to be greater than 1.8. Further the RNA Integrity Number (RIN) was analyzed using the Agilent Bioanalzyer. The samples needed to have RIN values of 7–10 and with a range of 1–1.5.

### Data analysis

We used 3 biological replicates in each group. The groups were: (1) Room air-WT, (2) Room air-*Cyp1a1*−/−, (3) Room air-*Cyp1a2*−/−, (4) Hyperoxia-WT, (5) Hyperoxia-*Cyp1a1*−/− and (6) Hyperoxia-*Cyp1a2*−/−.

## Microarray Data Analyses

The *Lumi* package^82^ implemented in the R statistical software^83^, version 2.14.1, was used to perform quality control of the signal intensity data on the transcript probes, background adjustment, variance stabilization transformation, and rank invariant normalization. A detection *p* value cutoff of 0.01 was required for the normalized intensities to consider a transcript as detected. Differentially Expressed Genes (DEG) were selected following the t-test comparing the groups of interest. The genes were considered to be differentially expressed for fdr-adjusted *q*-value < 0.2 and the linear fold chang ≥1.25 or <0.8. The volcano plots highlighting the DEGs were generated based on log2 fold change and –log10 p-values for each comparison, using the R statistical system. A graphical representation of the DEGs was generated in form of heatmaps of mean-centered normalized expression values and employing the euclidean distance metric the and average clustering method; the R statistical software was used for heatmap generation.

### Pathway enrichment and Transcription Factor Analysis

Rank file for each comparison was created based on the log2 fold change for each gene. We next employed Gene Set Enrichment Analysis (GSEA) methodology^27^ and software, against the Molecular Signature database (MSigDB) compendium of gene sets^84^. Gene Set Enrichment Analysis first finds an aggregate gene set score (termed enrichment score/ES) then runs 1000 permutations to establish a background distribution for ES. The ratio between ES and the average ES is termed Normalized Enrichment Score (NES). GSEA essentially determines whether a key component of a pathway or biological process gene set is significantly enriched in up-regulated genes (NES > 0, fdr-adjusted Q-value < 0.25) or in down-regulated genes (NES < 0, fdr-adjusted Q-value < 0.25). An established and fertile paradigm for hypothesis generation is that if the NES for a pathway in comparisons stemming from two different treatments are significant but having opposite signs, then the treatments might direct the pathways in opposite directions. The pathway collections KEGG, Reactome, Hallmark, and GOBP (Gene Ontology Biological Processes) were used to determine enriched pathways. We also used a compendium of putative transcription factor targets based on the TRANSFAC database to identifying enriched transcription factors in the transcriptome footprints analyzed. Overrepresentation (ORA) method was used to identify the key transcription factors modulating gene expression in our experiment (hypergeometric distribution; p < 0.05).

### RPPA profiling and analysis

Reverse phase protein array analysis (RPPA) was performed in the Functional Proteomics RPPA core facility at the MD Anderson Cancer Center. In brief, cell lysates were serially diluted by increasing two-fold ratios for 5 successive dilutions (ranging from undiluted to 1:16 dilution) and arrayed on nitrocellulose coated slides in an 11 × 11 format. Samples were probed with antibodies by tyramide-based signal amplification approach and visualized by DAB colorimetric reaction. Slides were scanned on a flatbed scanner to produce 16-bit tiff image. Spots from tiff images were identified and the density was quantified by Array-Pro Analyzer. Overall protein expression was normalized by integrating all dilution curves for all samples and all antibodies, using the Supercurve algorithm, implemented in the R statistical system^85^. Normalized linear values were analyzed for differential proteins following the same comparison scheme as in microarray dataset. Significance was assessed using a parametric t-test followed by Benjamini-Hochberg multiple hypothesis testing correction; we considered changes significant for Q < 0.25. A graphical representation in form of heatmaps for significantly changed proteins was generated using mean centering of the data and hierarchical clustering based on the Euclidean distance in the R statistical system. For a uniform visual representation of the RPPA results heatmap, expression of each protein were z-score transformed; e.g. for each protein, values were first mean-centered across all samples, then further divided by the standard deviation across all samples.

### Real time qPCR validation

A subset of genes was validated by quantitative real-time PCR (qRT-PCR) to validate the microarray results. We selected genes from the pool of DEGs in which were differentially regulated among the three genotypes. RNA (50 ng), isolated as above, was subjected to quantitative TaqMan RT-PCR using 7900HT Fast Real-Time PCR System (Applied Biosystems, Foster City, CA). Gene-specific primers purchased from life science technologies (Table S7). 18S was used as the reference gene. Quantitative values were obtained from the threshold PCR cycle number (Ct) at which the increase in signal was associated with an exponential growth for PCR product becomes detectable. Relative mRNA levels for chosen target genes were normalized to 18S content. Relative expression levels of each target gene were calculated according to the equation, 2^−ΔcT^, where ΔcT = Ct _target gene_ − Ct _18S gene_.

#### Western Blot analysis

For western blotting, lung whole protein (20 μg of protein) from individual male animals (n = 4/group) exposed to room air or to hyperoxia for 48 h, was prepared) as mentioned in the RPPA section and protein concentration was measured using the. Primary antibody against Rad51 (Cell Signaling; Cat#8875S; dilution 1:1000) and ERCC-1 (Santa Cruz; Cat#sc-17809; dilution 1:300) were purchased. Primary antibodies were used at a concentration of 1:1000 and secondary antibody at a concentration of 1:3000. β-actin was used as the loading control, followed by electrochemical detection of bands. The statistical analysis of densitometric values was done using Students t-test and p value < 0.05 was considered significant.

#### Measurement and analysis of lung oxidative DNA adducts

DNA (10 μg) was enzymatically degraded to normal (Np) and modified (Xp) deoxyribonucleoside 3′-monophosphates, as well as dinucleotides containing 3′-terminal cA (Np-cAp) with micrococcal nuclease and spleen phosphodiesterase at pH 6.0 and 37 °C for 3.5 h. After treatment of the mixture with nuclease P1 to specifically convert the four normal Np’s to nucleosides, the modified mononucleotides (Xp) or dinucleotides (Np-cAp) were converted to 5′-^32^P-labeled derivatives (pXp or pNp-cAp) by incubation with carrier-free [γ-^32^P]ATP and polynucleotide kinase. Chromatographic conditions were as previously described^28, 29^. Briefly, radioactively labeled digests were applied to modified PEI-cellulose thin layers and chromatographed overnight (15–16 h) with solvent 1 (2.8 M sodium phosphate, pH 5.2) (D1), to purify bulky adducts. Labeled adducts retained in the lower (L, 2.8 × 1.0 cm) and central (C, 2.8 × 1.0 cm) sections of the D1 chromatogram were briefly autoradiographed on Cronex 4 X-ray film and then contact-transferred to individual acceptor sheets and resolved by two-dimensional TLC. The non-polar L fractions were separated with solvents 2 (2.12 M lithium formate, 3.75 M urea, pH 3.35) and 3 (0.4 M sodium phosphate, 0.25 M Tris-HCl, 4.25 M urea, pH 8.2), and the polar C fractions with solvents 2 and 4(1.0 M sodium phosphate, pH 6.0), in the first and second dimensions, respectively. An additional development in the second dimension was performed with solvent 5 (1.0 M NaH2PO4, pH 6) in order to reduce background on C maps. ^32^P-labeled I-compounds were visualized by screen-enhanced autoradiography at −80 °C using Kodak XAR-5 films or with the aid of an InstantImager (Perkin Elmer, formerly Packard Instruments). Radioactivities of TLC fractions from individual animals were determined with the aid of an InstantImager. Appropriate blank count rates were automatically subtracted by the instrument from sample values. The extent of covalent DNA modification was estimated by calculating relative adduct labeling (RAL) values from corrected sample count rates, the amount of DNA assayed (expressed as pmol DNA monomer units or DNA-P), and the specific activity of [γ-^32^P]ATP according to

$${\rm{RAL}}=\frac{{\rm{DNA}}\,{\rm{modification}}({\rm{s}})\,[{\rm{cpm}}]}{\mathrm{DNA} \mbox{-} {\rm{P}}\,[{\rm{pmol}}]\times {\rm{Spec}}{\rm{.}}\,{\rm{act}}{.}_{{\rm{ATP}}}[{\rm{cpm}}/{\rm{pmol}}]}$$

Quantitative data represented minimum estimates because 100% adduct recovery presumably was not achieved.

## Electronic supplementary material

Supplementary Info
