# Supplementary material for: Role of Cytochrome P450 (CYP)1A in Hyperoxic Lung Injury: Analysis of the Transcriptome and Proteome
Source: Sci Rep. 2017 Apr 4;7:642. doi: 10.1038/s41598-017-00516-x (PMC5428698; doi:10.1038/s41598-017-00516-x)
Supplement: Supplementary file 1 — Supplementary Info [file 41598_2017_516_MOESM1_ESM.pdf]

Supplementary Information

Title: Role of Cytochrome P450 (CYP)1A in Hyperoxic Lung Injury: Analysis of the Transcriptome and Proteome

Authors: Krithika Lingappan[a], Suman Maity[b], Weiwu Jiang[a], Lihua Wang[a], Xanthi Couroucli[a], Alex Weith[a,c], Guodong Zhou[d], Cristian Coarfa[b] and Bhagavatula Moorthy[a*]

Table S1: Up regulated genes across all genotypes (Hyperoxia exposed mice compared to room air controls)

WT		
**Gene Symbol**	**Gene Name**	**Fold Change**
GDF15	Growth Differentiation Factor 15	19.85
NUPR1	Nuclear Protein 1, Transcriptional Regulator	13.41
MT1	Metallothionein 1	12.71
CDKN1A	Cyclin Dependent Kinase Inhibitor 1A	8.01
AKAP12	A-Kinase Anchoring Protein 12	7.10
ANKRD1	Ankyrin Repeat Domain 1	6.49
TNFRSF12A	TNF Receptor Superfamily Member 12A	6.36
CTGF	Connective Tissue Growth Factor	5.47
GADD45G	Growth Arrest And DNA Damage Inducible Gamma	5.47
Cyp1a1-/-		
**Gene Symbol**	**Gene Name**	**Fold Change**
GDF15	Growth Differentiation Factor 15	18.19
NUPR1	Nuclear Protein 1, Transcriptional Regulator	11.31
ANKRD1	Ankyrin Repeat Domain 1	10.22
CDKN1A	Cyclin Dependent Kinase Inhibitor 1A	6.53
MT1	Metallothionein 1	5.79
TGM2	Transglutaminase 2	5.49
TRIB3	Tribbles Pseudokinase 3	5.26
TNFRSF12A	TNF Receptor Superfamily Member 12A	5.13
Cyp1a2-/-		
**Gene Symbol**	**Gene Name**	**Fold**

		Change
GDF15	Growth Differentiation Factor 15	18.49
NUPR1	Nuclear Protein 1, Transcriptional Regulator	15.19
ANKRD1	Ankyrin Repeat Domain 1	8.63
AREG	Amphiregulin	7.72
MT1	Metallothionein 1	6.75
TNFRSF12A	TNF Receptor Superfamily Member 12A	5.92
MTHFD2	Methylenetetrahydrofolate Dehydrogenase (NADP+ Dependent) 2, Methenyltetrahydrofolate Cyclohydrolase	5.16
ASNS	Asparagine Synthetase (Glutamine-Hydrolyzing)	5.13
CTGF	Connective Tissue Growth Factor	5.04

Table S2: Down regulated genes across all genotypes (Hyperoxia exposed mice compared to room air controls)

WT		
**Gene Symbol**	**Gene Name**	**Fold Change**
ASAH3L	N-Acylsphingosine Amidohydrolase 3-Like	0.26
EAR2	V-Erba-Related Protein 2	0.23
EDNRB	Endothelin Receptor Type B	0.24
FMO1	Flavin-Containing Monooxygenase	0.28
LYZ1	Lysozyme (Renal Amyloidosis)	0.26
MUP2	Methionine Permease	0.24
TMEM100	Transmembrane Protein 100	0.22
Cyp1a1-/-		
**Gene Symbol**	**Gene Name**	**Fold Change**
ASAH3L	N-Acylsphingosine Amidohydrolase 3-Like	0.28
CXCL12	C-X-C Motif Chemokine Ligand 12	0.29
EAR2	V-Erba-Related Protein 2	0.22
LYZ1	Lysozyme (Renal Amyloidosis)	0.22
MUP2	Methionine Permease	0.26
TMEM100	Transmembrane Protein 100	0.29
Cyp1a2-/-		
**Gene Symbol**	**Gene Name**	**Fold Change**
EAR2	V-Erba-Related Protein 2	0.17
EAR3	V-Erba-Related Protein 3	0.20

LYZ1	Lysozyme (Renal Amyloidosis)	0.25
SFTPA1	Surfactant Protein A1	0.26
THBD	Thrombomodulin	0.28
TMEM100	Transmembrane Protein 100	0.19

Table S4: Down regulated genes in Room Air (WT vs. *Cyp1a1-/-* and WT vs. *Cyp1a2-/-)*

Cyp1a1-/- vs. WT		
**Gene Symbol**	**Gene Name**	**Fold Change**
CBX1	Chromobox 1	0.19
CD200	CD200	0.28
RPL29	Ribosomal protein L29	0.29
NNMT	Nicotinamide N-methyltransferase	0.34
EPM2AIP1	EPM2A (laforin) interacting protein 1	0.40
MELA	Melanoma antigen	0.54
ATG3	Autophagy related 3	0.55
CLASRP	CLK4-associating serine/arginine rich protein	0.55
WDR82	WD repeat domain containing 82	0.55
CD59	CD59	0.55
Cyp1a2-/- vs. WT		
**Gene Symbol**	**Gene Name**	**Fold Change**
MUP2	Major urinary protein 2	0.26
MUP3	Major urinary protein 3	0.38
TTR	Transthyretin	0.47
MUP5	Major urinary protein 5	0.48
APOA	Apolipoprotein A-II	0.51
RHBDL2	Rhomboid, veinlet-like 2	0.52
ACTC1	Actin, alpha, cardiac muscle 1	0.54
KLF2	Kruppel like factor 2	0.58
MT-ND5	NADH dehydrogenase 5, mitochondrial	0.59

Table S3: Up regulated genes in Room Air (WT vs. *Cyp1a1-/-* and WT vs. *Cyp1a2-/-*)

***Cyp1a1-/-* vs. WT**		
**Gene Symbol**	**Gene Name**	**Fold Change**
ERDR1	Erythroid Differentiation Regulator 1	3.21
PGLYRP1	Peptidoglycan recognition protein 1	2.4
ADAT2	Adenosine deaminase, tRNA specific 2	1.89
SORL1	Sortilin related receptor 1	1.75
Rpl29-ps2	Ribosomal protein L29, pseudogene 2	1.62
SLC27A6	Solute carrier family 27 (fatty acid transporter), member 6	1.53
HSPA8	Heat shock protein 8	1.78
KLF2	Kruppel-like factor 2	1.65
IL18	Interleukin-18	1.58
***Cyp1a2-/-* vs. WT**		
**Gene Symbol**	**Gene Name**	**Fold Change**
MAP3K6	Mitogen-activated protein kinase kinase kinase 6	2.32
KLF9	Kruppel-like factor 9	2.14
ANGPTL4	Angiopoietin-like 4	2.05
FKBP5	FK506 binding protein 5	2.01
FAM107A	Family with sequence similarity 107, member A	1.93
NPR3	Natriuretic peptide receptor 3	1.92
EAR3	Eosinophil-associated, ribonuclease A family, member 3	1.87
SPARC	Secreted acidic cysteine rich glycoprotein	1.85
LPL	Lipoprotein lipase	1.82

Figure S1

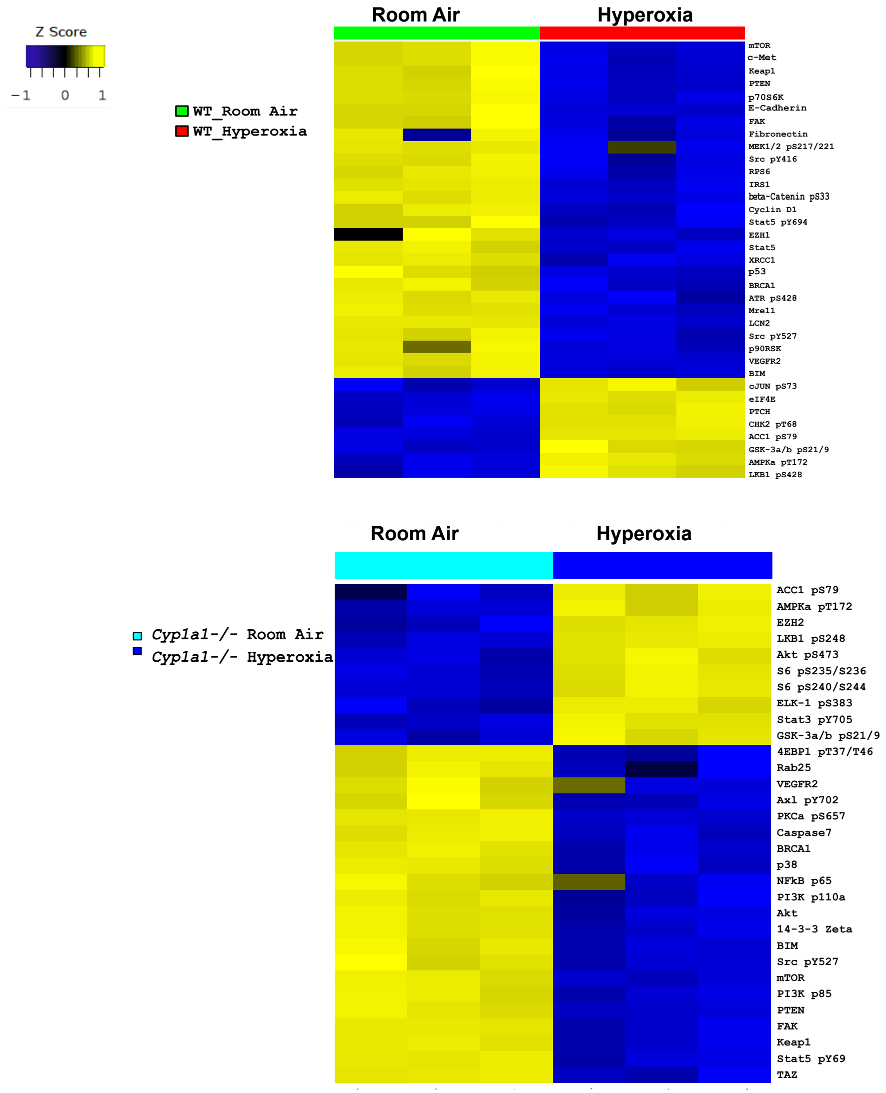

Figure S1. Hyperoxia-exposed *WT* and *Cyp1a1-/-* mice are characterized by robust and distinct proteomic changes compared to room air controls. Reverse Phase Protein Analysis (RPPA) was used to generate proteomic profiles of WT and *Cyp1a1-/-* mice exposed to hyperoxia. Differential protein expression was evaluated using parametric t-test (significance for fdr-adjusted q-value<0.25). Proteomic changes of hyperoxia exposed mice compared to room air controls in WT and *Cyp1a1-/-* mice.

Table S5: RPPA profile of differentially expressed proteins in the lung after exposure to hyperoxia in *Cyp1a1-/-* and *Cyp1a2-/-* compared to WT mice

**Antibody**	**Gene Symbol**	***Cyp1a1-/-* over WT fold change**	***Cyp1a1-/-* over WT p-value**	***Cyp1a1-/-* over WT fdr-adjusted q-value**	**Antibody**	**Gene Symbol**	***Cyp1a2-/-* over WT fold change**	***Cyp1a2-/-* over WT p-value**	***Cyp1a2-/-* over WT fdr-adjusted q-value**
AMPKa pT172	PRKAA1	0.65	0.01	0.08	c-Raf	RAF1	0.73	0.00	0.10
GSK-3a/b pS21/9	GSK3A, GSK3B	0.65	0.03	0.18	Stat5	STAT5	0.74	0.00	0.10
CHK2 pT68	CHEK2	0.68	0.00	0.01	IGF1R	IGF1R	0.79	0.01	0.15
ELK-1 pS383	ELK1	0.72	0.01	0.08	ACC1	ACACA	0.81	0.00	0.10
PTCH	PTCH1	0.74	0.01	0.07	mTOR	FRAP1	0.85	0.01	0.13
Stat3 pY705	STAT3	0.74	0.03	0.18	VEGFR2	KDR	0.88	0.01	0.15
LKB1 pS428	STK11	0.75	0.00	0.01	Bcl-xl	BCL2L1	1.27	0.00	0.10
eIF4E	EIF4E	0.87	0.00	0.01	p70S6K pT389	RPS6KB1	1.34	0.01	0.15
Bcl-xl	BCL2L1	0.89	0.03	0.18	Stat3 pY705	STAT3	1.43	0.00	0.13
Stat5 pY694	STAT5	1.07	0.06	0.25	S6 pS240/S244	RPS6	1.58	0.02	0.23
GSK3B	GSK3B	1.14	0.05	0.23					
PI3K p110a	PIK3CA	1.14	0.01	0.08					
p38	MAPK14	1.15	0.01	0.10					
E-Cadherin	CDH1	1.15	0.00	0.02					
PI3K p85	PIK3R1	1.19	0.00	0.04					
Src pY527	SRC	1.23	0.01	0.10					
4EBP1 pT37/T46	EIF4EBP1	1.25	0.03	0.16					
p70S6K	RPS6KB1	1.26	0.01	0.10					
c-Met	MET	1.29	0.05	0.23					
ACC1	ACACA	1.31	0.04	0.19					

EGFR pY1173	EGFR	1.32	0.05	0.23					
mTOR	FRAP1	1.32	0.00	0.01					
Keap1	KEAP1	1.32	0.00	0.01					
PKCa	PRKCA	1.33	0.02	0.12					
14-3-3 Zeta	YWHAZ	1.39	0.00	0.05					
beta-Catenin pS33	CTNNB1	1.40	0.02	0.10					
PKCa pS657	PRKCA	1.45	0.00	0.02					
VEGFR2	KDR	1.62	0.01	0.08					
BRCA1	BRCA1	1.68	0.00	0.04					
PTEN	PTEN	1.71	0.00	0.01					
LCN2	LCN2	1.85	0.05	0.23					
Mre11	MRE11A	2.04	0.00	0.04					

Table S6: RPPA profile of differentially expressed proteins in the lung after exposure in WT and *Cyp1a1-/-* mice after exposure to hyperoxia compared to room air (RA) controls.

Antibody	Gene Symbol	WT O_2 over WT RA fold change	WT O_2 over WT RA p-value	WT O_2 over WT RA fdr-adjusted q-value	Antibody	Gene Symbol	*Cyp1a1-/-* O_2 over *Cyp1a1-/-* RA fold change	*Cyp1a1-/-* O_2 over *Cyp1a1-/-* RA p-value	*Cyp1a1-/-* O_2 over *Cyp1a1-/-* RA fdr-adjusted q-value
LCN2	LCN2	0.45	0.00	0.057	Caspase7	CASP7	0.63	0.01	0.091
Mre11	MRE11A	0.47	0.00	0.058	BIM	BCL2L11	0.75	0.01	0.117
VEGFR2	KDR	0.65	0.01	0.125	BRCA1	BRCA1	0.77	0.01	0.091
PTEN	PTEN	0.72	0.02	0.141	PKCa pS657	PRKCA	0.79	0.00	0.059
BRCA1	BRCA1	0.72	0.02	0.131	Stat5 pY694	STAT5	0.80	0.01	0.117
c-Met	MET	0.76	0.02	0.133	PTEN	PTEN	0.81	0.01	0.091
Src pY416	SRC	0.77	0.03	0.191	Keap1	KEAP1	0.82	0.01	0.091
p70S6K	RPS6KB1	0.77	0.01	0.122	VEGFR2	KDR	0.83	0.04	0.226
E-Cadherin	CDH1	0.77	0.06	0.247	p38	MAPK14	0.84	0.02	0.159
beta-Catenin pS33	CTNNB1	0.79	0.00	0.034	Akt	AKT1 AKT2 AKT3	0.85	0.01	0.117
p53	TP53	0.79	0.05	0.231	14-3-3 Zeta	YWHAZ	0.85	0.01	0.091
Src pY527	SRC	0.79	0.01	0.122	4EBP1 pT37/T46	EIF4EBP1	0.85	0.04	0.226
BIM	BCL2L11	0.81	0.03	0.162	Src pY527	SRC	0.86	0.04	0.226
Stat5	STAT5	0.81	0.01	0.125	Axl pY702	AXL	0.87	0.05	0.244
Keap1	KEAP1	0.83	0.04	0.191	PI3K p85	PIK3R1	0.88	0.01	0.091
mTOR	FRAP1	0.83	0.03	0.162	FAK	PTK2	0.88	0.02	0.147
RPS6	RPS6	0.84	0.01	0.122	mTOR	FRAP1	0.90	0.01	0.091
XRCC1	XRCC1	0.84	0.01	0.122	PI3K p110a	PIK3CA	0.90	0.05	0.226

p90RSK	RPS6KA1	0.87	0.04	0.197	NFkB p65	NFKB	0.93	0.05	0.235
IRS1	IRS1	0.88	0.01	0.106	TAZ	WWTR1	0.93	0.02	0.153
ATR pS428	ATR	0.88	0.03	0.162	Rab25	RAB25	0.94	0.05	0.228
Fibronectin	FN1	0.89	0.05	0.243	EZH2	EZH2	1.09	0.04	0.226
Cyclin D1	CCND1	0.89	0.02	0.131	ELK-1 pS383	ELK1	1.17	0.03	0.159
FAK	PTK2	0.90	0.06	0.247	ACC1 pS79	ACACA ACACB	1.22	0.05	0.226
Stat5 pY694	STAT5	0.90	0.06	0.247	GSK-3a/b pS21/9	GSK3A, GSK3B	1.22	0.01	0.112
EZH2	EZH2	0.92	0.06	0.247	LKB1 pS428	STK11	1.23	0.00	0.078
MEK1/2 pS217/221	MAP2K1	0.93	0.06	0.247	Stat3 pY705	STAT3	1.32	0.00	0.091
LKB1 pS428	STK11	1.10	0.02	0.161	Akt.pS473	AKT1 AKT2 AKT3	1.39	0.01	0.091
eIF4E	EIF4E	1.14	0.00	0.057	AMPKa pT172	PRKAA1	1.56	0.02	0.149
cJUN pS73	JUN	1.23	0.03	0.171	S6 pS235/S236	RPS6	1.67	0.00	0.091
PTCH	PTCH1	1.26	0.01	0.089	S6 pS240/S244	RPS6	2.10	0.01	0.091
CHK2 pT68	CHEK2	1.31	0.00	0.089					
GSK-3a/b pS21/9	GSK3A, GSK3B	1.40	0.05	0.231					
AMPKa pT172	PRKAA1	2.14	0.00	0.061					
ACC1 pS79	ACACA ACACB	2.22	0.00	0.006					

Table S7: Primer List

	Gene Symbol	Gene name	Identifier
1	Ankrd1	Ankyrin repeat domain 1	Mm00496512_m1
2	Gdf15	Growth differentiation factor 15	Mm00442228_m1
3	Nupr1	Nuclear protein transcription regulator 1	Mm00498104_m1
4	Areg	Amphiegulin	Mm01354339-m1
5	P21(Trp63)	Transformation protein63	Mm00495793-m1
6	CTGF	Connective tissue growth factor	Mm01192933-g1
7	Hmox1	Heme oxygenase1	Mm00516005-m1
8	Dusp1	daul specificity phosph	Mm00457274-g1
9	Slc2a3	solutate carrier family2 member3	Mm00441483-m1
10	Nme1	Nucleoside diphosphate kines1	Mm01612215-m1
11	PCNA	Proliferating cell nuclear antigen	Mm00448100-g1
12	XPC	Xeroderma pigmentosum complementation groupC	Mm01183434-m1
13	CD63	CD63 antigen	Mm01966817-g1
14	Abca1	ATP-binding casette sub-family A (ABC1),member1	Mm00442646-m1
